# Supplementary material for: A single-cell transcriptional atlas reveals resident progenitor cell niche functions in TMJ disc development and injury
Source: Nat Commun. 2023 Feb 14;14:830. doi: 10.1038/s41467-023-36406-2 (PMC9929076; doi:10.1038/s41467-023-36406-2)
Supplement: Supplementary file 1 — Supplementary information [file 41467_2023_36406_MOESM1_ESM.pdf]

Supplementary information

for

‘A single-cell transcriptional atlas reveals resident progenitor cell niche functions in TMJ disc development and injury’

Ruiye Bi[1*], Qing Yin[1, 2*], Haohan Li[1*], Xianni Yang[1], Yiru Wang[1], Qianli Li[1], Han Fang[1], Peiran Li[1], Ping Lyu[3], Yi Fan[3], Binbin Ying[4], Songsong Zhu[1#]

[1] State Key Laboratory of Oral Diseases, National Clinical Research Center for Oral Diseases, Department of Orthognathic and TMJ Surgery, West China Hospital of Stomatology, Sichuan University, Chengdu, 610041, China

[2] Max-Planck Institute for Heart and Lung Research, W. G. Kerckhoff Institute, Bad Nauheim, D-61231, Germany

[3] State Key Laboratory of Oral Diseases, National Clinical Research Center for Oral Diseases, Department of Operative Dentistry and Endodontics, West China Hospital of Stomatology, Sichuan University, Chengdu, 610041, China

[4] Department of Stomatology, Ningbo First Hospital, 59 Liuting street，Ningbo 315000, China

***These authors contribute equally to this work**

# Corresponding author: Songsong Zhu,

E-mail: ZSS_1977@163.com; Postal address: Department of Orthognathic and TMJ Surgery, No. 14, 3rd Section of Ren Min Nan Rd, West China Hospital of Stomatology, Sichuan University, Chengdu, 610041, China

Supplementary Figure 1

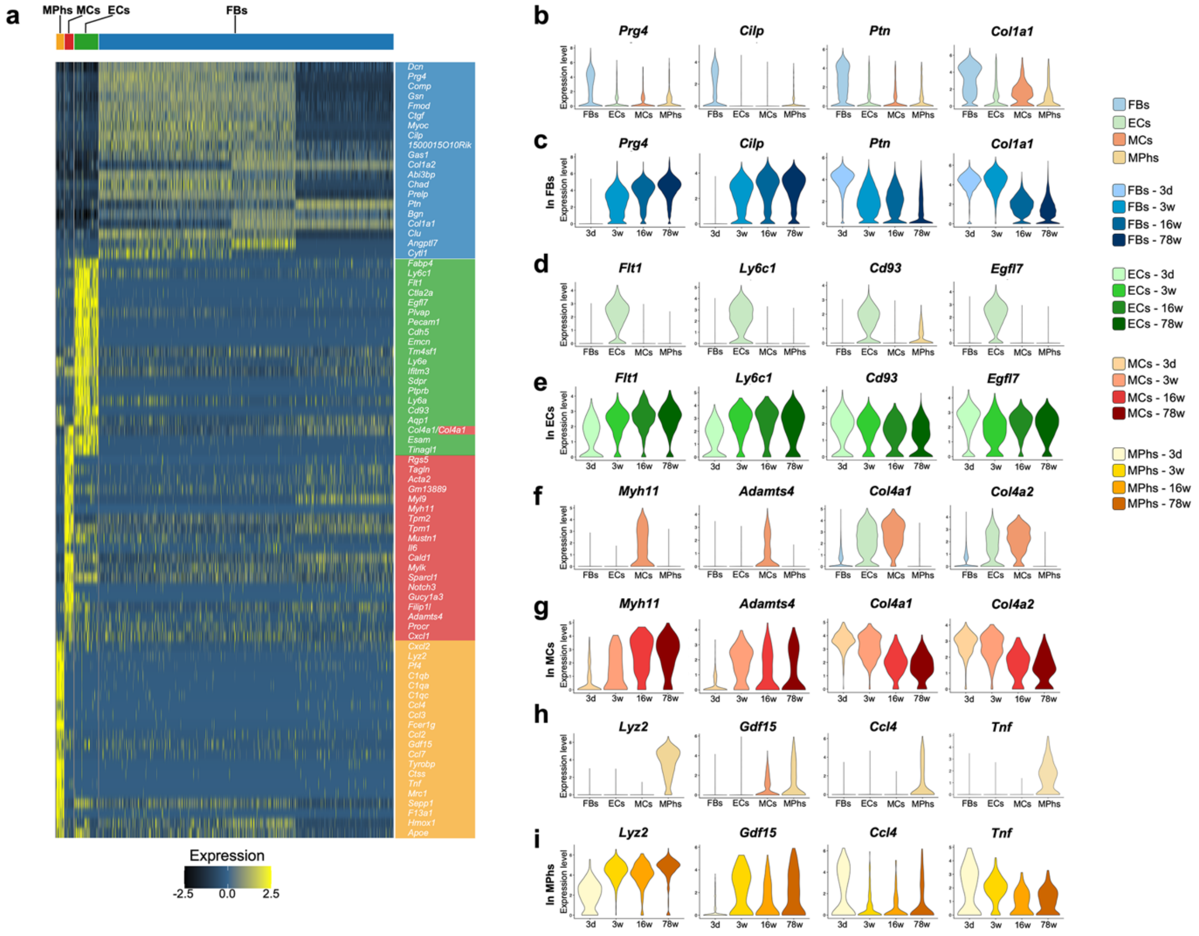

Figure S1: The four principal cell types in TMJ discs have marked expression variations. (a) Heatmap of the top 20 genes of the 4 main cell types in TMJ discs. Expression values are normalized and scaled averages. Blue: fibroblasts (FBs); yellow: macrophages (MPhs); green: endothelial cells (ECs); and red: mural cells (MCs). **(b)** Violin plot of the expression of selected differentially expressed genes (DEGs) of FB clusters in the four principal cell types. Expression values are nonnormalized. **(c)** Violin plot of the expression of selected differentially expressed genes (DEGs) of FB clusters at different stages (3 d, 3 w, 16 w, and 78 w). Expression values are nonnormalized. **(d)** Violin plot of the expression of selected differentially expressed genes (DEGs) in the EC cluster in the four principal cell types. Expression values are nonnormalized. **(e)** Violin plot of the expression of selected differentially expressed genes (DEGs) in the EC cluster at different stages (3 d, 3 w, 16 w, and 78 w). Expression values are nonnormalized. **(f)** Violin plot of the expression of selected differentially expressed genes (DEGs) of the MC cluster in the four principal cell types. Expression values are nonnormalized. **(g)** Violin plot of the expression of selected differentially expressed genes (DEGs) of the MC cluster at different stages (3 d, 3 w, 16 w, and 78 w). Expression values are nonnormalized. **(h)** Violin plot of the expression of selected differentially expressed genes (DEGs) of the MPh cluster in the four principal cell types. Expression values are nonnormalized. **(i)** Violin plot of the expression of selected differentially expressed genes (DEGs) of the MPh cluster at different stages (3 d, 3 w, 16 w, and 78 w). Expression values are nonnormalized.

Supplementary Figure 2

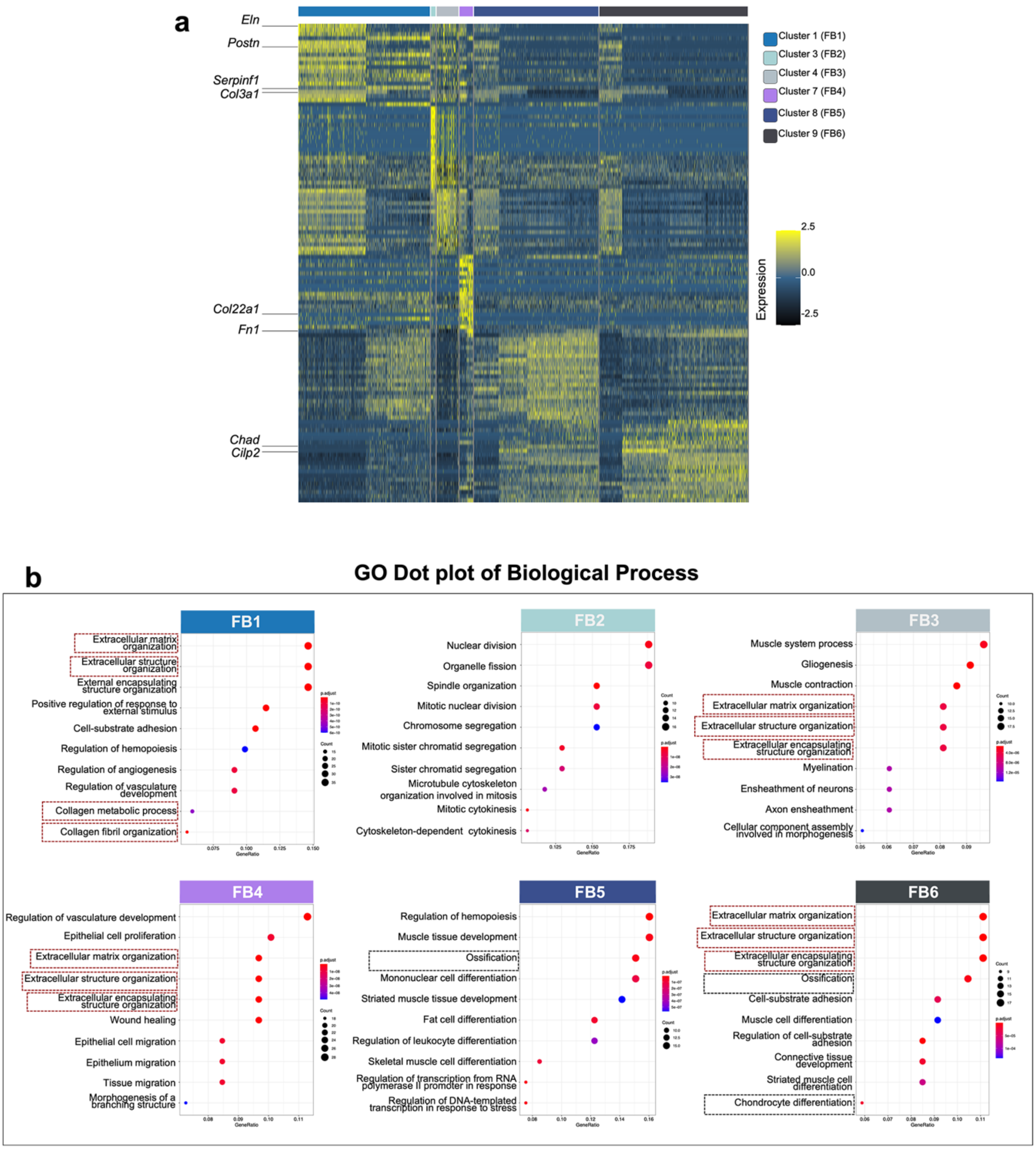

Figure S2: Extracellular matrix related genes are highly expressed in fibroblast clusters. (a) Heatmap of the top 20 DEGs of FB clusters with selected extracellular matrix (ECM) genes marked. ECM-related genes are highly expressed in FB clusters. Expression values are normalized and scaled averages. **(b)** Dot plot of the biological processes of different FB clusters. Gene Set Enrichment Analysis was used to determine p value using GO (biological process) database on FBs. We emphasized the novel GO terms related to FB function.

Supplementary Figure 3

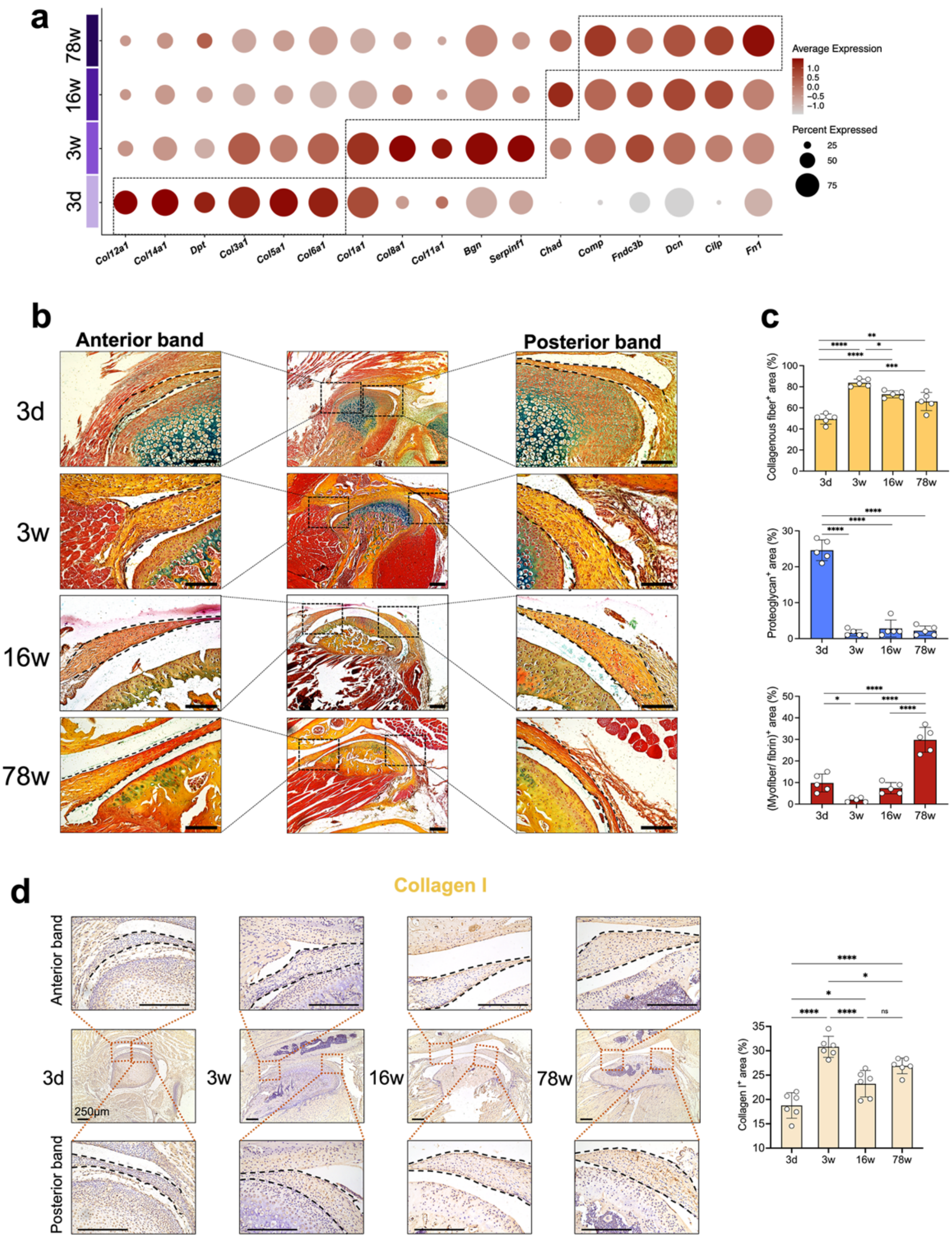

Figure S3: Expressions of extracellular matrix markers and components change through different postnatal stages. (a) Extracellular matrix (ECM) markers of FBs at different stages. Expression values are normalized and scaled averages. **(b)** Cross-sections and longitudinal sections of the mouse TMJ. Movat pentachrome staining shows changes in extracellular substances at different stages (3 d, 3 w, 16 w, and 78 w). A: anterior band, P: posterior band, white dotted lines: boundary of TMJ discs. Experiments were performed using 5 independent animals at each stage with similar results. Scale bar: 200μm. **(c)** Semi-quantification of ECM component expression *via* Movat pentachrome staining. Data are presented as mean values +/- SD. The one-way ANOVA with Tukey's multiple comparison test was used for data analysis. N = 5 independent animals at each stage. *(3w vs. 16w (collagenous fiber$^+$ area%)) p = 0.0283, *(3d vs. 3w (myofiber/fibrin$^+$ area%)) p = 0.0241, **(3d vs.

78w (collagenous fiber$^+$ area%)) p = 0.0011, **(3w vs. 78w (collagenous fiber$^+$ area%)) p = 0.0006, ****p<0.0001. Yellow: collagen fiber; blue: glycoprotein; and red: myofiber/fibrin. **(d)** Collagen I immunohistochemical staining of TMJ discs showing the distribution of collagen I at different stages. Black dotted line: boundary of discs, scale bar = 250 μm. Collagen I expression was evaluated by immunohistochemistry and the percentage of Collagen I$^+$ area in the TMJ disc was quantified using ImageJ. Data are presented as mean values +/- SD. N = 6 independent animals. The one-way ANOVA with Tukey's multiple comparison test was used for data analysis, *(3d vs. 16w) p = 0.0164, *(3w vs. 78w) p = 0.0385, ****p < 0.0001.

Supplementary Figure 4

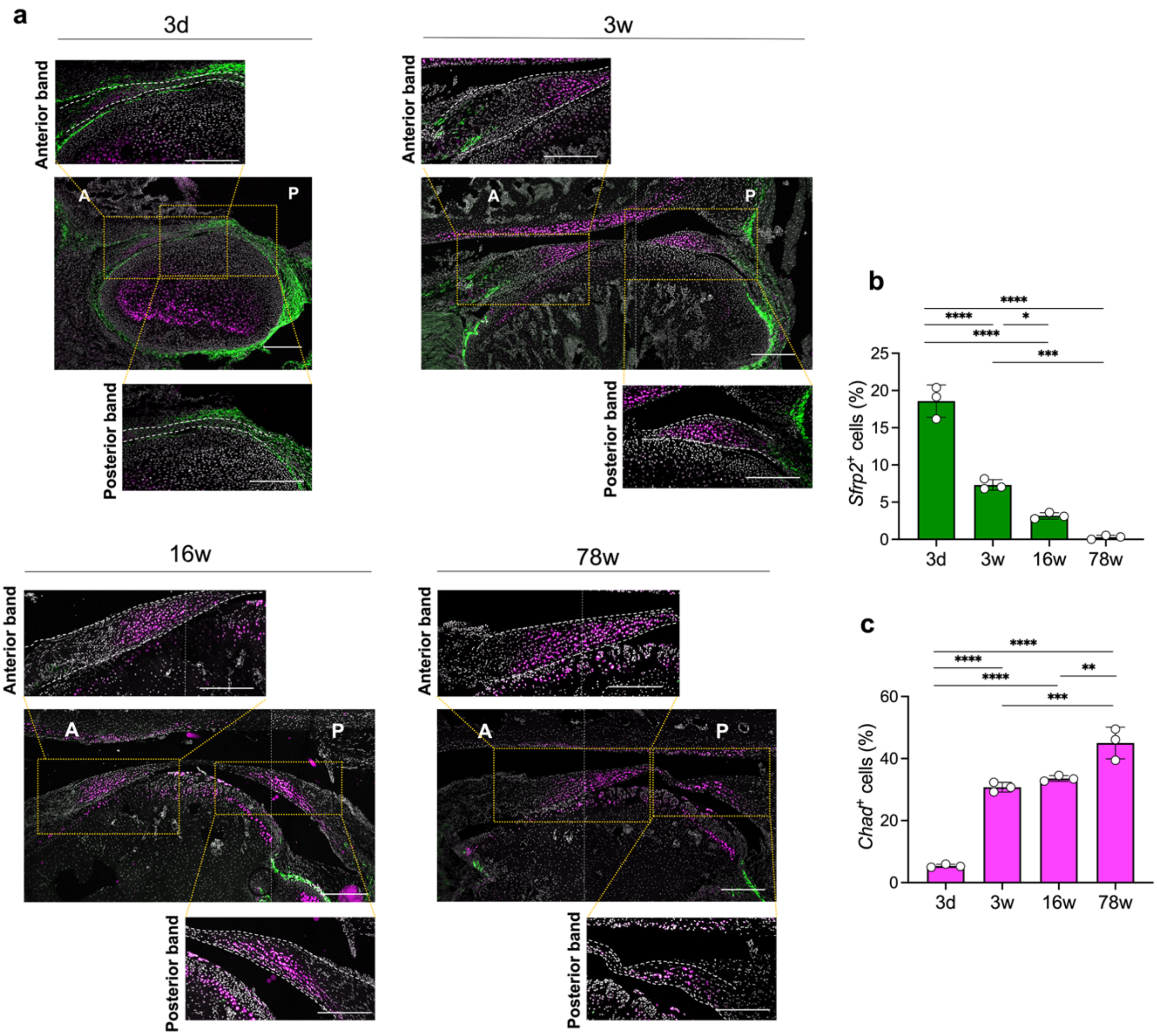

Figure S4: Spatial-temporal expression heterogeneity of chondrogenic and non-chondrogenic FBs
(a) Fluorescence in situ hybridization of the disc for *Sfrp2* and *Chad*. Green: *Sfrp2*; magenta: *Chad*; gray: DAPI. A: anterior band, P: posterior band, white dotted lines: boundary of the discs. scale bar: 200 µm. **(b)** *Sfrp2*$^{+}$ cell numbers were semi-quantified using ImageJ 1.51. Data are presented as mean+/-SD. N=3 independent animals. The one-way ANOVA with Tukey's multiple comparison test was used, *p=0.0105, ***p=0.0004, ****p<0.0001.**(c)** *Chad*$^{+}$ cell numbers were semi-quantified using ImageJ 1.51. Data are presented as mean+/-SD. N=3 independent animals. The one-way ANOVA with Tukey's multiple comparison test was used, **p=0.0038, ***p=0.0009, ****p<0.0001.

Supplementary Figure 5

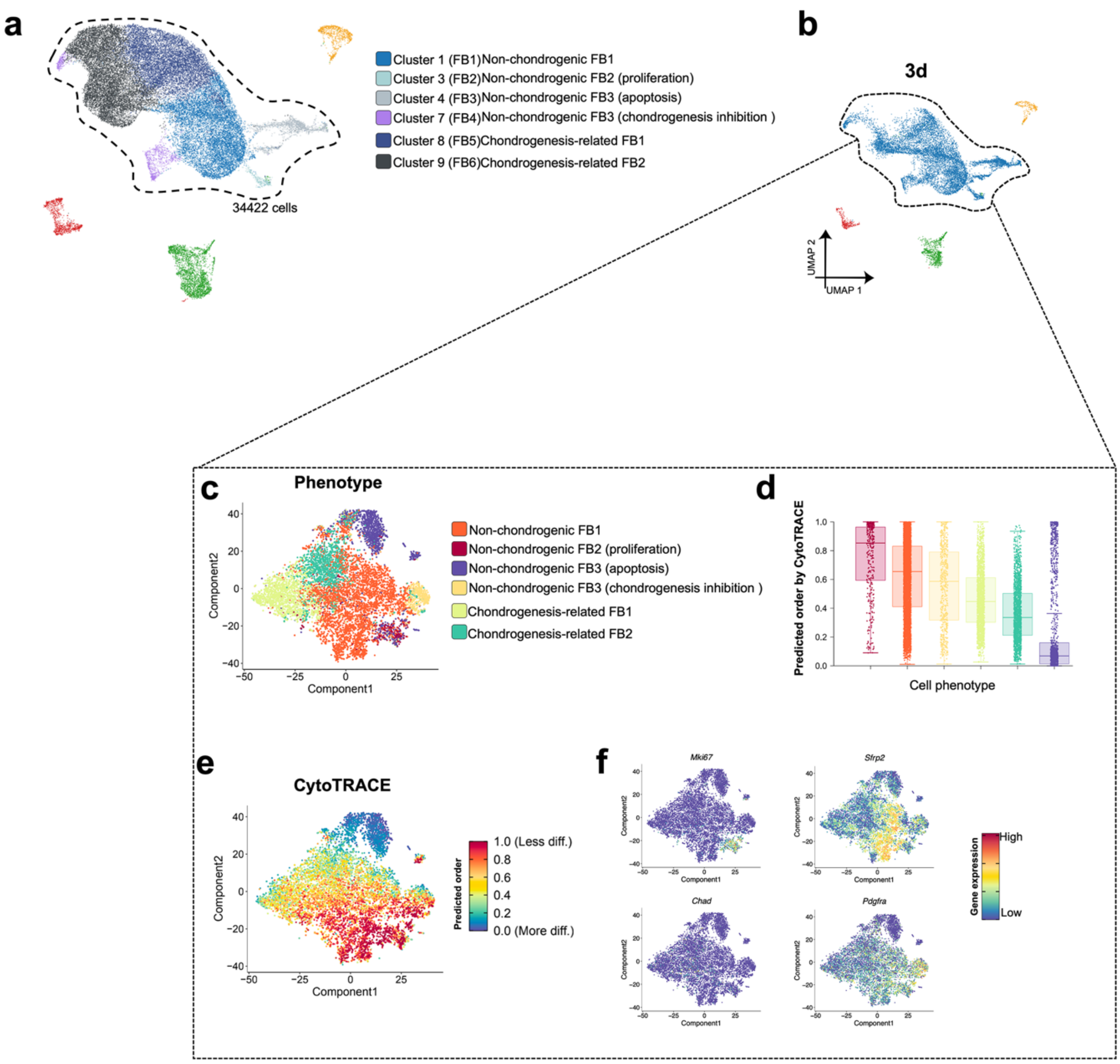

Figure S5: Chondrogenesis related fibroblast clusters are associated with disc aging and injury. (a) UMAP of fibroblast clusters with re-defined cluster name of FBs. Clusters were generated using a resolution of 0.2 prior to subclustering into major cell types according to the Methods. **(b)** UMAP of fibroblast clusters at d3. **(c)** CytoTRACE analysis of FBs at the d3 stage. **(d)** Boxplots showing CytoTRACE values for FBs at d3 stage. N (FB1) = 5238 cells, n (FB2) = 346 cells, n (FB3) = 1573 cells, n (FB4) = 559 cells, n (FB5) = 1942 cells, n (FB6) = 1760 cells. **(e)** FBs coloured by developmental potential order. **(f)** FBs coloured by expression values of different markers.

Supplementary Figure 6

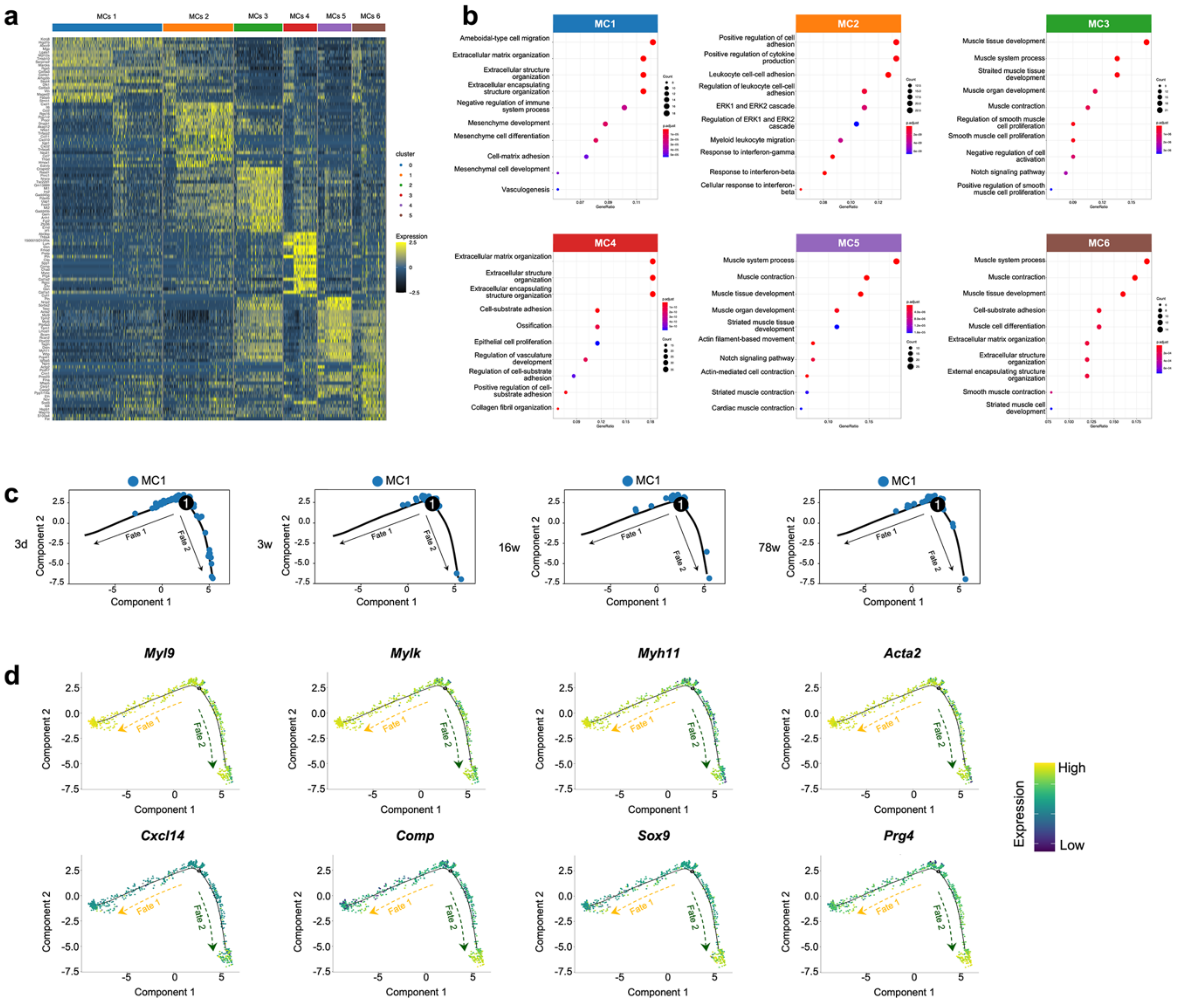

Figure S6: Mural cell clusters show heterogeneity with distinct pseudotime lineage fates involving either self-renewal or functional differentiation. (a) Heatmap of the top 20 DEGs of reclustered MC subclusters. Expression values are normalized and scaled averages. **(b)** Dot plot of the biological processes of different MC subclusters. 6 MC subclusters were annotated according to their GO terms related to skeletal progenitor and smooth muscle functions. Gene Set Enrichment Analysis was used to determine p value using GO (biological process) database on MCs. **(c)** Monocle analysis of MC subclusters at different stages. **(d)** Pseudotime Monocle analysis of the indicated selected genes from the root of the trajectory to fate 1 and fate 2. Bar color indicates selected gene expression levels.

Supplementary Figure 7

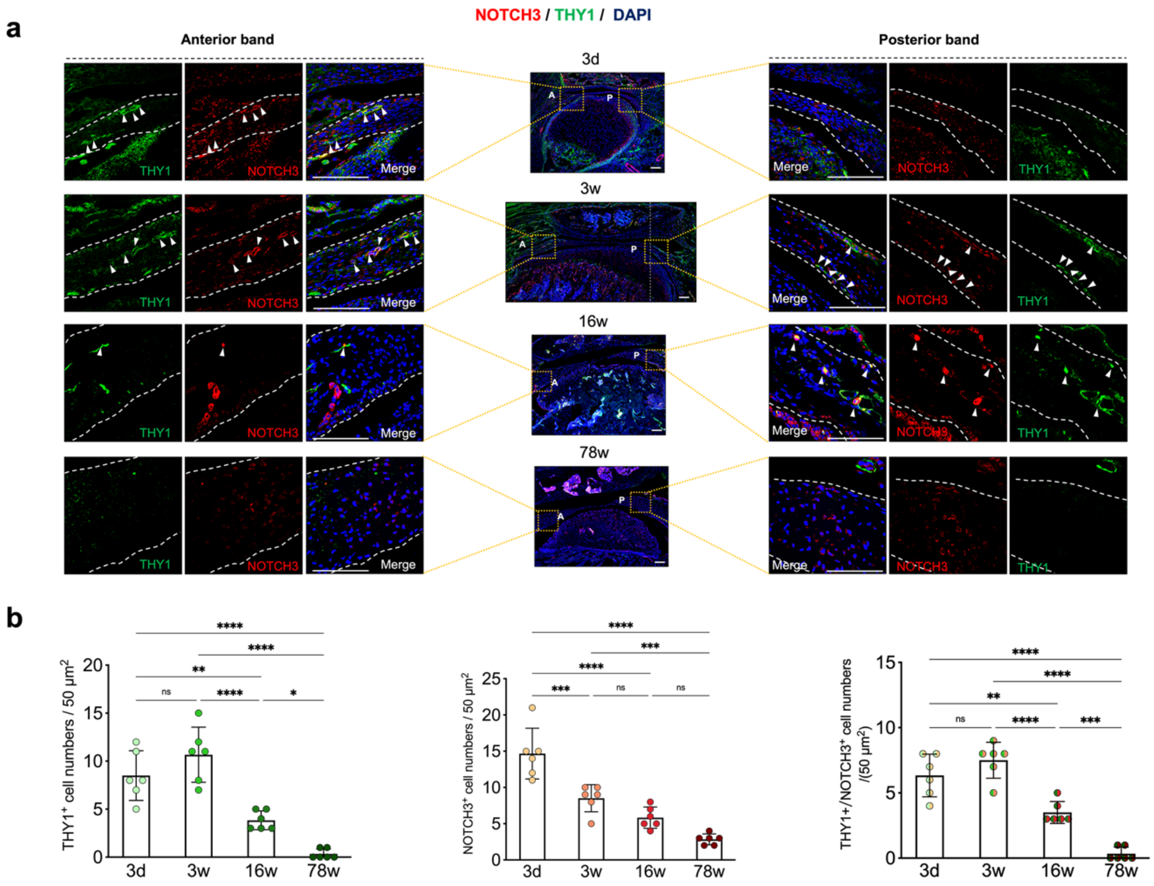

Figure S7: Expression pattern changes of NOTCH3 and THY1 in TMJ disc at different postnatal stages. (a) Immunofluorescence staining of TMJ discs at different postnatal stages for NOTCH3 and THY1. Green: THY1; red: NOTCH3; and blue: DAPI. A: anterior band, P: posterior band, white dotted lines: boundary of the discs. White triangular arrows: double-positive cells, scale bar: 50 μm. **(b)** $THY1^+$, $NOTCH3^+$ and $THY1^+$/ $NOTCH3^+$ cell numbers from 3 d, 3 w, 16 w and 78 w mice were quantified using ImageJ 1.51. Data are presented as mean values +/- SD. N = 6 independent animals. The one-way ANOVA with Tukey's multiple comparison test was used for data analysis, *(16w vs. 78w ($THY1^+$ cell number)) $p = 0.0321$, **(3d vs. 16w ($THY1^+$ cell number)) $p = 0.0035$, **(3d vs. 16w ($THY1^+/NOTCH3^+$ cell number)) $p = 0.0024$, ***(3d vs. 3w ($NOTCH3^+$ cell number)) $p = 0.0004$, ***(3w vs. 78w ($NOTCH3^+$ cell number)) $p = 0.0010$, ***(16w vs. 78w ($THY1^+/NOTCH3^+$ cell number)) $p = 0.0008$, ****$p < 0.0001$.

Supplementary Figure 8

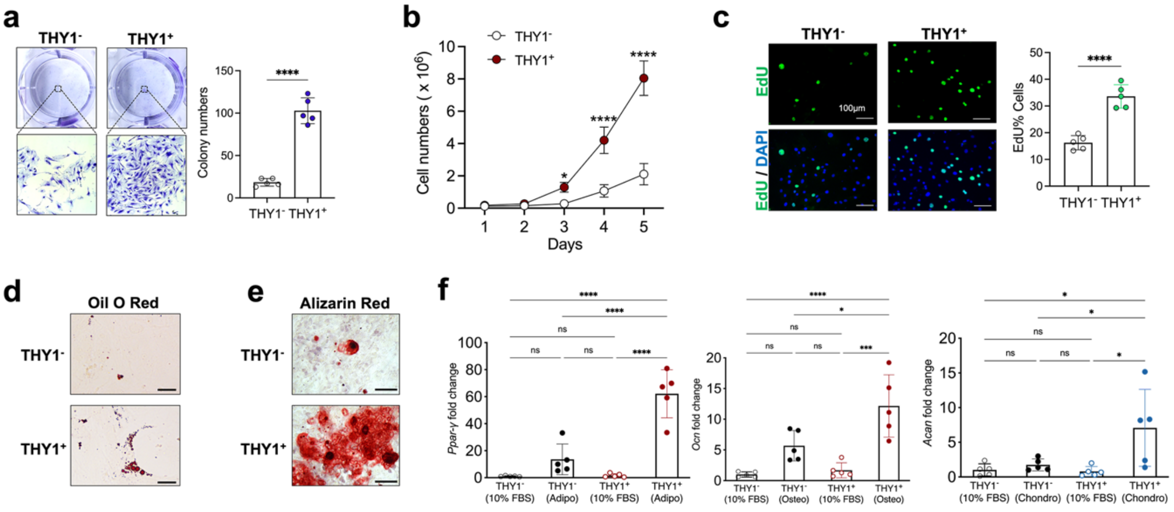

Figure S8: THY1^{+} MCs show progenitor capacity *in vitro*. (a) Colony formation assay of THY1^{+}/ THY1^{-} cells. N=5 biological replicates, data are presented as mean+/-SD. The two tailed t test was used for analysis, ****p $<$ 0.0001. **(b)** Cell growth curve of THY1^{+}/THY1^{-} disc cells. N=5 biological replicates. Data are presented as mean+/-SD. The two-way ANOVA with Tukey's multiple comparison test was used for data analysis, *p=0.0279, ****p $<$ 0.0001. **(c)** EdU: slow-cycling cells (green). DAPI: cell nuclei (blue). N=5 biological replicates, data are presented as mean+/-SD. The two tailed t test was used for analysis, ****p$<$0.0001. Scale bar=50 µm. **(d)** Oil red O staining of THY1^{+}/THY1^{-} cells. N=3 biological replicates. Scale bar=100 µm. **(e)** Alizarin red staining of THY1^{+}/THY1^{-} cells. N=3 biological replicates. Scale bar= 500 µm. **(f)** qRT-PCR in THY1^{+}/THY1^{-} cells. Data are mean fold change of *Ppar-γ*, *Ocn* and *Acan*, normalized to *Gapdh* (mean+/-SD). N=5 independent experiments; The one-way ANOVA with Tukey's multiple comparison test was used for data analysis, *(THY1^{-}(Osteo) vs. THY1^{+}(Osteo)) p=0.0131, *(THY1^{-}(10% FBS) vs. THY1^{+}(Chondro)) p=0.0192, *(THY1^{+}(10% FBS) vs. THY1^{+}(Chondro)) p=0.0149, *(THY1^{-}(Chondro) vs. THY1^{+}(Chondro)) p=0.0420, ***p=0.0002, ****p$<$0.0001.

Supplementary Figure 9

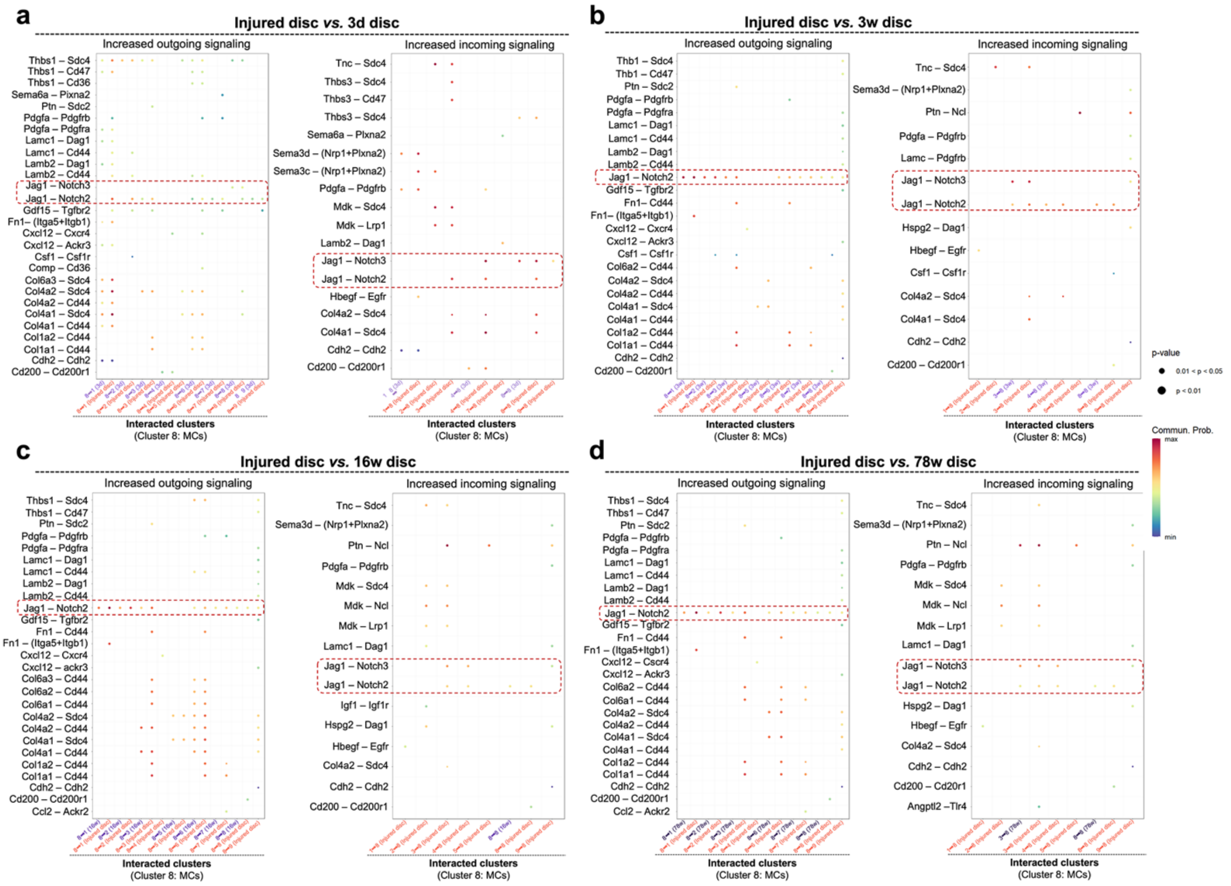

Figure S9: The signaling pathways involved in cell interactions between different cell clusters in injured TMJ discs. (a) Increased outgoing and incoming MC-related receptor-ligand signaling communication probabilities in injured TMJ disc MCs in comparison with 3d disc MCs. **(b)** Increased outgoing and incoming MC-related receptor-ligand signaling communication probabilities in injured TMJ disc MCs in comparison with 3w disc MCs. **(c)** Increased outgoing and incoming MC-related receptor-ligand signaling communication probabilities in injured TMJ disc MCs in comparison with 16w disc MCs. **(d)** Increased outgoing and incoming MC-related receptor-ligand signaling communication probabilities in injured TMJ disc MCs in comparison with 78w disc MCs.

Supplementary Figure 10

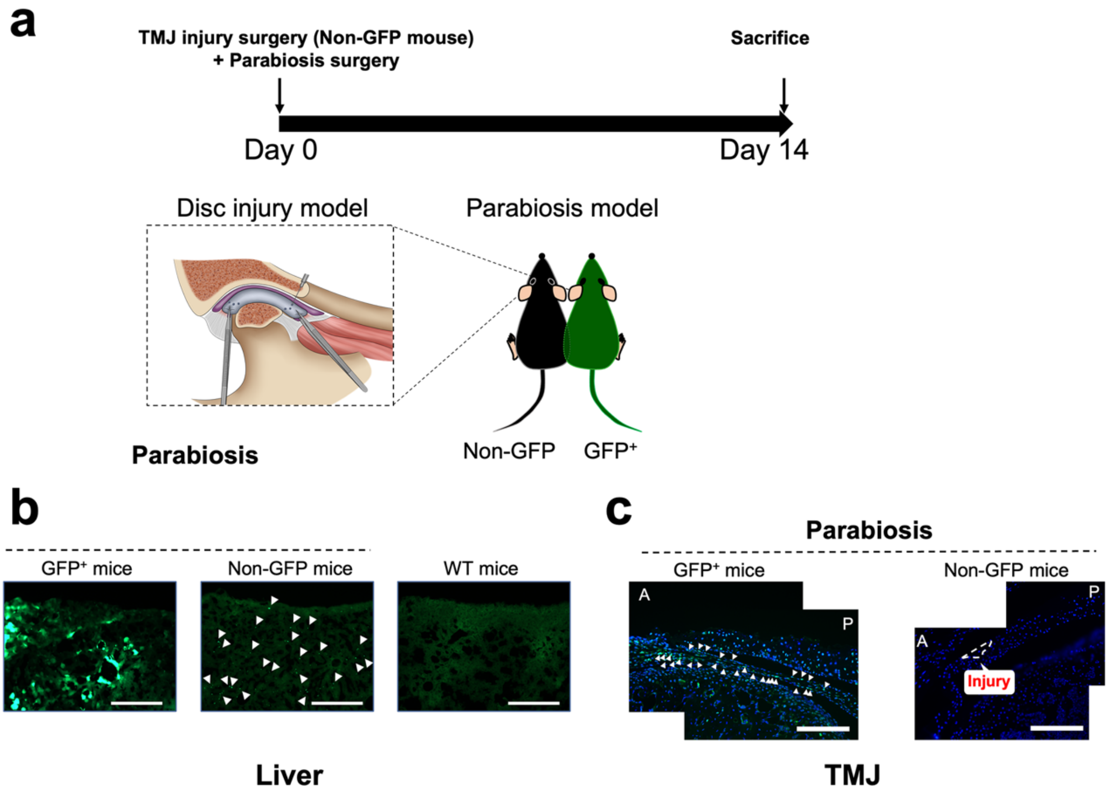

Figure S10: Circulating cells are not the main cell source participating in joint disc injury and repair. (a) Schematic diagram of surgically induced TMJ injury in a parabiosis model with WT mice and GFP mice. In the parabiosis model, a GFP^{+} mouse was paired with a non-GFP mouse that then underwent damage-inducing surgery on the right TMJ. Two weeks later, the TMJs and livers of the parabiosis pairs were harvested for further analysis. (n=3). **(b)** GFP in the livers of GFP^{+} and non-GFP mice from the parabiosis model and from individual wild-type mice. Cross-sections of livers were observed under a fluorescence microscope. White triangular arrows show GFP^{+} cells, Experiments were performed using 3 independent animals in each group with similar results. Scale bar=200 μm. **(c)** GFP in the TMJ discs of GFP^{+} and non-GFP mice from the parabiosis model. Cross-sections of TMJ discs were observed under a fluorescence microscope. White dotted line: injury area, scale bar = 200 μm.

Supplementary Figure 11

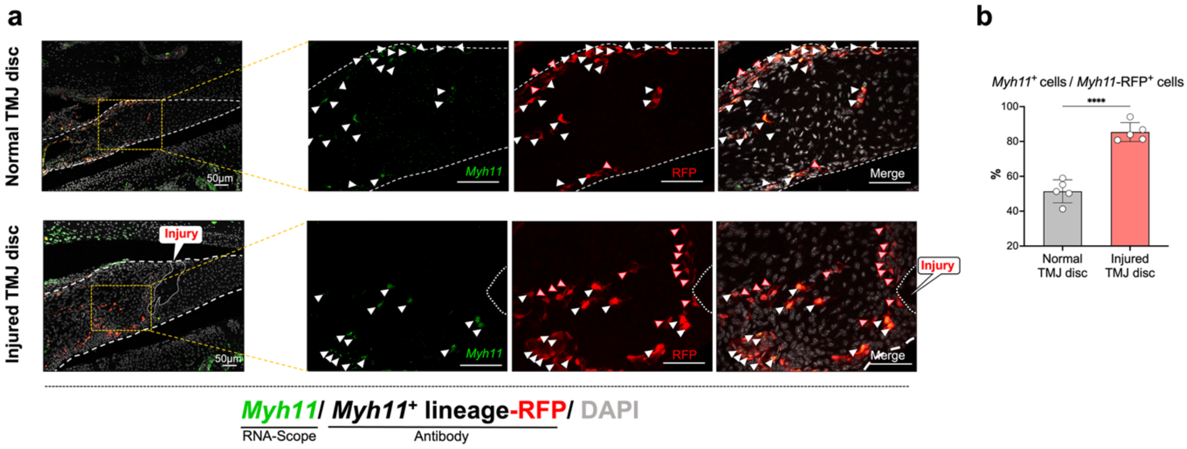

Figure S11: *Myh11*$^{+}$ lineage cells migrate to impaired region upon TMJ disc injury. (a) Fluorescence in situ hybridization of normal and injured TMJ discs for *Myh11* combined with spontaneous *Myh11*-Cre-driven red fluorescence. As MC lineage migrated to middle band, lower expression of *Myh11* was observed. Green: *Myh11*; Red: *Myh11*$^{+}$ lineage-RFP; gray: DAPI. A: anterior band, white dotted lines: boundary of the discs. Scale bar: 50 μm. **(b)** Semi-quantification of the ratio between *Myh11*$^{+}$ lineage-RFP / *Myh11* double positive cells and *Myh11*$^{+}$ lineage-RFP cells in normal disc and injured disc. ****$p < 0.0001$. Data are presented as mean values +/- SD. N = 5 independent animals in each group. The two tailed t test was used for data analysis. ****$p < 0.0001$.

Supplementary Figure 12

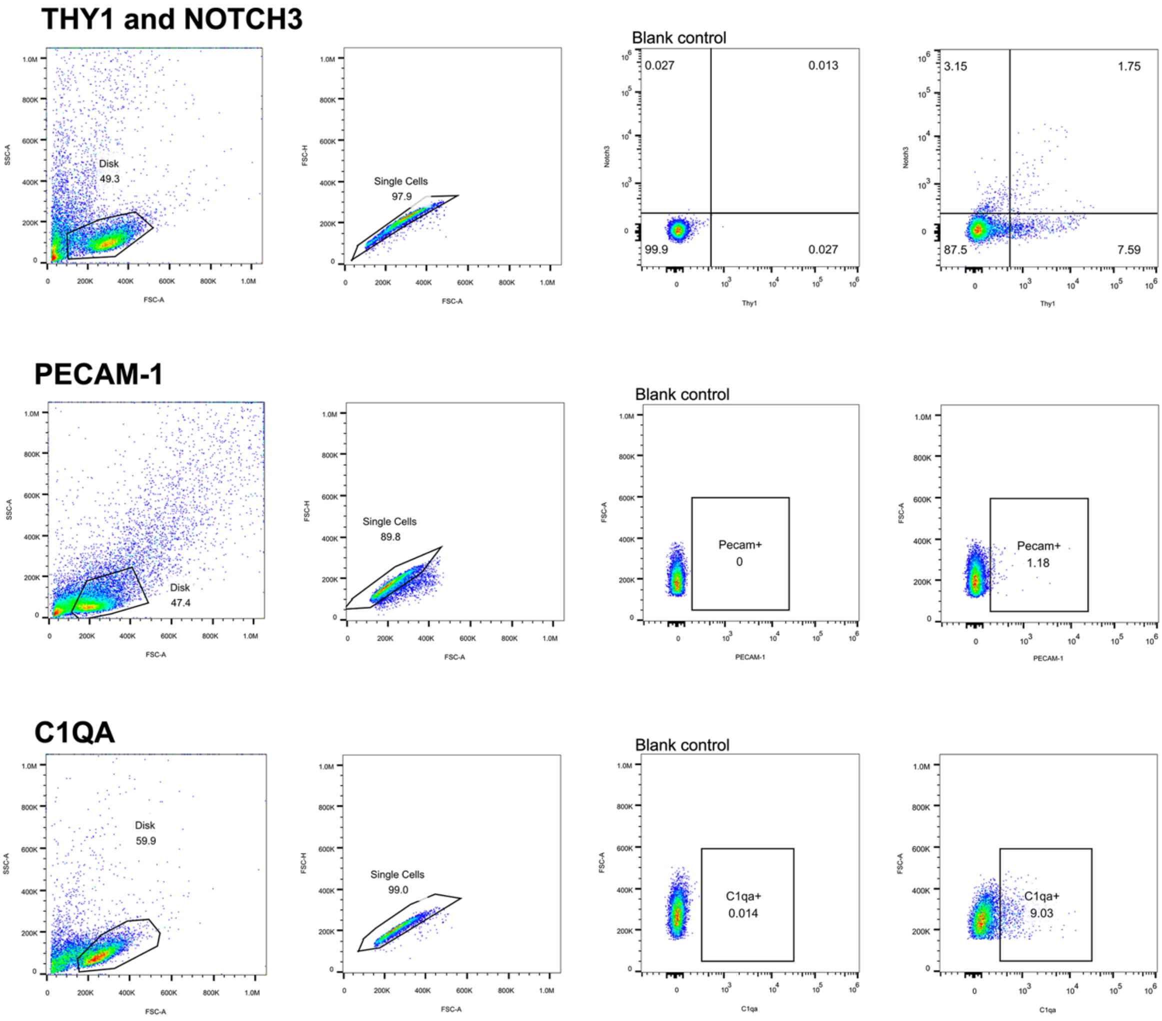

Figure S12: Gating strategies of the flow cytometry. The FSC vs. SSC gating strategy is used to exclude cell debris and dead cells, which tends to have lower forward scatter levels and are found at the bottom left corner of the FSC vs. SSC density plot. Apoptotic cells tend to have lower FSC and higher SSC. FCS-A vs. FSC-H gating strategy is used to exclude doublets and to screen out single cells. Each test has a blank control which is used to screen out positive cells.
