# Supplementary material for: A single-cell transcriptional atlas reveals resident progenitor cell niche functions in TMJ disc development and injury
Source: Nat Commun. 2023 Feb 14;14:830. doi: 10.1038/s41467-023-36406-2 (PMC9929076; doi:10.1038/s41467-023-36406-2)
Supplement: Supplementary file 3 — Description of Additional Supplementary Files [file 41467_2023_36406_MOESM3_ESM.pdf]

Description of Additional Supplementary Files

File Name: Supplementary Movie 1
Description: Z-stack immunofluorescence of RFP/PECAM1/NOTCH3 analysis of the anterior band of normal TMJ disc. Green: Pecam1; Red: *Myh11*$^{+}$ lineage-RFP; Yellow: NOCTH3; blue: DAPI.

File Name: Supplementary Movie 2
Description: Z-stack immunofluorescence of RFP/PECAM1/NOTCH3 analysis of the anterior band of injured TMJ disc. Green: Pecam1; Red: *Myh11*$^{+}$ lineage-RFP; Yellow: NOCTH3; blue: DAPI.

File Name: Supplementary Movie 3
Description: Z-stack immunofluorescence of RFP/THY1/NOTCH3 analysis of the anterior band of normal TMJ disc. Green: THY1; Red: *Myh11*$^{+}$ lineage-RFP; Yellow: NOCTH3; blue: DAPI.

File Name: Supplementary Movie 4
Description: Z-stack immunofluorescence of RFP/THY1/NOTCH3 analysis of the anterior band of injured TMJ disc. Green: THY1; Red: *Myh11*$^{+}$ lineage-RFP; Yellow: NOCTH3; blue: DAPI.
