# Supplementary material for: A single-cell transcriptional atlas reveals resident progenitor cell niche functions in TMJ disc development and injury
Source: Nat Commun. 2023 Feb 14;14:830. doi: 10.1038/s41467-023-36406-2 (PMC9929076; doi:10.1038/s41467-023-36406-2)
Supplement: Supplementary file 8 — Reporting Summary [file 41467_2023_36406_MOESM8_ESM.pdf]

nature portfolio

DBPR
Corresponding author(s): Songsong Zhu
Last updated by author(s): Jan 24, 2023

Reporting Summary

Nature Portfolio wishes to improve the reproducibility of the work that we publish. This form provides structure for consistency and transparency in reporting. For further information on Nature Portfolio policies, see our Editorial Policies and the Editorial Policy Checklist.

Statistics

For all statistical analyses, confirm that the following items are present in the figure legend, table legend, main text, or Methods section.

n/a	Confirmed	
☐	☒	The exact sample size (*n*) for each experimental group/condition, given as a discrete number and unit of measurement
☐	☒	A statement on whether measurements were taken from distinct samples or whether the same sample was measured repeatedly
☐	☒	The statistical test(s) used AND whether they are one- or two-sided<br>*Only common tests should be described solely by name; describe more complex techniques in the Methods section.*
☒	☐	A description of all covariates tested
☐	☒	A description of any assumptions or corrections, such as tests of normality and adjustment for multiple comparisons
☐	☒	A full description of the statistical parameters including central tendency (e.g. means) or other basic estimates (e.g. regression coefficient) AND variation (e.g. standard deviation) or associated estimates of uncertainty (e.g. confidence intervals)
☐	☒	For null hypothesis testing, the test statistic (e.g. *F*, *t*, *r*) with confidence intervals, effect sizes, degrees of freedom and *P* value noted<br>*Give P values as exact values whenever suitable.*
☒	☐	For Bayesian analysis, information on the choice of priors and Markov chain Monte Carlo settings
☒	☐	For hierarchical and complex designs, identification of the appropriate level for tests and full reporting of outcomes
☒	☐	Estimates of effect sizes (e.g. Cohen's *d*, Pearson's *r*), indicating how they were calculated

Our web collection on statistics for biologists contains articles on many of the points above.

Software and code

Policy information about availability of computer code

Data collection	There was no software used for data collection
Data analysis	FCM was performed using a flow cytometer (AttuneTM NxT Flow Cytometer Thermo Fisher Scientific, USA), and analysis was performed using FlowJo: Flow Cytometry Analysis Software, v10.6.2 (Tree Star).<br>Single-cell digestion and sequencing: In brief, the discs were disolated precisely under a stereomicroscope. Next, TMJ discs were digested into cell suspension. After filtering, the cell suspension was loaded into Chromium microfluidic chips with 3' chemistry and barcoded with a 10x Chromium Controller. RNA from the barcoded cells was subsequently reverse-transcribed, and sequencing libraries were constructed with reagents from a Chromium Single Cell 3' v2 reagent kit according to the manufacturer's instructions. Sequencing was performed with an Illumina system. Cell Ranger (version 3.1.0) was used to process the raw data and generate the UMI matrix. Count files for each condition were read into RStudio, and datasets corresponding to each sample were labeled. All downstream analysis were performed with Seurat (v 3.0.1) in the R environment (version 3.6.1). Only cells found to express more than 200 transcripts were considered to limit contamination from dead or dying cells. To remove potential batch effects, FindIntegrationAnchors and IntegrateData functions were used to integrate datasets.<br>The data were then scaled and central features in the dataset were identified using ScaleData function, and PCA components were used for an initial clustering of the cells (using RunPCA function). The top 30 PCs were used to perform UMAP analysis. The clusters were then refined by using the FindClusters function. The clusters were annotated by manual review of the marker genes of each individual cluster.<br>Stained sections were visualized with a laser scanning confocal microscope (LSCM) (Olympus FV3000, Japan) or an optical microscope (Leica DM2000 & DM2000 LED, Germany).<br>All statistical analysis were finished using the GraphPad Prism 9 Software (GraphPad Software Inc., San Diego, CA, USA).

For manuscripts utilizing custom algorithms or software that are central to the research but not yet described in published literature, software must be made available to editors and reviewers. We strongly encourage code deposition in a community repository (e.g. GitHub). See the Nature Portfolio guidelines for submitting code & software for further information.

Data

Policy information about availability of data

All manuscripts must include a data availability statement. This statement should provide the following information, where applicable:

- Accession codes, unique identifiers, or web links for publicly available datasets
- A description of any restrictions on data availability
- For clinical datasets or third party data, please ensure that the statement adheres to our policy

For this study, our scRNA-seq datasets are available on GEO accession number GSE218785. Source data are provided with this paper.

ds

Human research participants

Policy information about studies involving human research participants and Sex and Gender in Research.

Reporting on sex and gender	*Use the terms sex (biological attribute) and gender (shaped by social and cultural circumstances) carefully in order to avoid confusing both terms. Indicate if findings apply to only one sex or gender; describe whether sex and gender were considered in study design whether sex and/or gender was determined based on self-reporting or assigned and methods used. Provide in the source data disaggregated sex and gender data where this information has been collected, and consent has been obtained for sharing of individual-level data; provide overall numbers in this Reporting Summary. Please state if this information has not been collected. Report sex- and gender-based analyses where performed, justify reasons for lack of sex- and gender-based analysis.*
Population characteristics	*Describe the covariate-relevant population characteristics of the human research participants (e.g. age, genotypic information, past and current diagnosis and treatment categories). If you filled out the behavioural & social sciences study design questions and have nothing to add here, write "See above."*
Recruitment	*Describe how participants were recruited. Outline any potential self-selection bias or other biases that may be present and how these are likely to impact results.*
Ethics oversight	*Identify the organization(s) that approved the study protocol.*

Note that full information on the approval of the study protocol must also be provided in the manuscript.

Field-specific reporting

Please select the one below that is the best fit for your research. If you are not sure, read the appropriate sections before making your selection.

☒ Life sciences ☐ Behavioural & social sciences ☐ Ecological, evolutionary & environmental sciences

For a reference copy of the document with all sections, see nature.com/documents/nr-reporting-summary-flat.pdf

Life sciences study design

All studies must disclose on these points even when the disclosure is negative.

Sample size	No statistical method was used to predetermine sample size.
Data exclusions	no data were excluded from the analyses.
Replication	All results represent the mean+/- SD from at least 3 independent biological samples . All the replication were successful.
Randomization	All the male/female mice were wild type mice selected from different litters randomly. Both male and female 3-day pups were used for one bulk single cell suspension. 3-week, 16-week and 78-week mice used for all the experiments were males. Since the transgenic locus is located on the Y chromosome, only male mice (Myh11-Cre^{ER}; Tm$^{fl/-}$) were used for MCs lineage tracing.
Blinding	Every quantification was measured by 2 investigators individually and blindly when positive cell numbers and positive staining area were counted.

Reporting for specific materials, systems and methods

We require information from authors about some types of materials, experimental systems and methods used in many studies. Here, indicate whether each material, system or method listed is relevant to your study. If you are not sure if a list item applies to your research, read the appropriate section before selecting a response.

Materials & experimental systems

n/a	Involved in the study
☐	☒ Antibodies
☒	☐ Eukaryotic cell lines
☒	☐ Palaeontology and archaeology
☐	☒ Animals and other organisms
☒	☐ Clinical data
☒	☐ Dual use research of concern

Methods

n/a	Involved in the study
☒	☐ ChIP-seq
☐	☒ Flow cytometry
☒	☐ MRI-based neuroimaging

Antibodies

Antibodies used	PolAlexa flour 647 monoclonal rabbit-anti NOTCH3 antibody (130512, Biolegend, USA).<br>Polyclonal rat-anti PECAM-1 antibody (550274, BD Pharmingen, USA).<br>Monoclonal mouse-anti C1QA antibody (NBP1-51139, Novus, USA).<br>Monoclonal rat-anti CD90.2 (14-0902-82, Invitrogen, USA).<br>Polyclonal rabbit-anti NOTCH3 (PA519515, Invitrogen, USA).<br>Polyclonal Goat anti-rat 488 (a23240, Abbkine, USA).<br>Polyclonal Goat anti-rabbit 568 (A-11036, Invitrogen, USA).<br>Polyclonal Goat anti-rabbit 647 (HA1123, Huabio, China).<br>Polyclonal Goat anti-mouse 488 (A32723, Invitrogen, USA).
Validation	The validation of each antibody used in this study was determined by manufactures. Species reactivity and applications could be found on their website by using catalog number below: https://www.biolegend.com/; https://www.bd.com/; https://www.thermofisher.com/; https://www.novusbio.com/; https://www.abcam.cn/; https://www.abbkine.cn/; http://www.huabio.cn/

Animals and other research organisms

Policy information about studies involving animals; ARRIVE guidelines recommended for reporting animal research, and Sex and Gender in Research

Laboratory animals	FVB-Tg(Myh11-cre/ERT2)1Soff/J mice (JAX#019079, abbreviated Myh11-CreER) and B6.Cg-Gt(ROSA)26Sortm14(CAG-tdTomato)Hze/J mice (JAX#07908, abbreviated Tmfl/fl) at the age of 7 weeks old were obtained from Jackson Laboratory. These mice were mated with 7-week-old C57/BL6 mice (Dashuo Experimental Animal Laboratories, Chengdu). To generate tdTomato-conditionally activated mice, Tmfl/fl mice were crossed with Myh11-CreERT mice. 8-week-old immunodeficient NOD-SCID mice (C001180, Cyagen Biosciences, China) were used as transplant recipients for renal capsule transplantation of prospective MC progenitor populations. 8-week-old GFP+ mice (C001180, Cyagen Biosciences, China) were used for generation of a parabiosis model with non-GFP mice. All animals were maintained in appropriate environment at 24°C with 40% humidity in a 12-hour light/dark cycle with free access to water and irradiated diet. Wood bedding and igloo covers were provided for environmental enrichment. Mice were fed a standard chow diet. All the male/female mice were wild type mice selected from different litters randomly. Both male and female 3-day pups were used for one bulk single cell suspension. 3-week, 16-week and 78-week mice used for all the experiments were males. Since the transgenic locus is located on the Y chromosome, only male mice (Myh11-CreER; Tmfl/-) were used for MCs lineage tracing.
Wild animals	This study dis not use wild animals.
Reporting on sex	Due to the specificity of lineage tracing of MCs, the transgenic locus is located on the Y chromosome, only male mice can be used for this experiments; otherwise, all animal experiments are free of sex differences.
Field-collected samples	This study did not involve samples collected from field.
Ethics oversight	Animal procedures were performed according to protocols approved by the Animal Ethics Committee of Sichuan University (WCHSIRB-D-2021-231).

Note that full information on the approval of the study protocol must also be provided in the manuscript.

Flow Cytometry

Plots

Confirm that:

☒ The axis labels state the marker and fluorochrome used (e.g. CD4-FITC).

☒ The axis scales are clearly visible. Include numbers along axes only for bottom left plot of group (a 'group' is an analysis of identical markers).

☒ All plots are contour plots with outliers or pseudocolor plots.

☒ A numerical value for number of cells or percentage (with statistics) is provided.

Methodology

Sample preparation	TMJ discs were isolated from C57BL/6 mice at different stages (3 d, 3 w, 16 w, and 78-82 w) and then cut into small pieces. Next, these small disc pieces were digested with pronkase for 1hour and collagenase P for 0.5-1 hour. The dissociated cells were centrifuged, filtered through nylon mesh, and resuspended in cell staining buffer.
Instrument	AttuneTM NxT Flow Cytometer Thermo Fisher Scientific, USA
Software	FlowJo: Flow Cytometry Analysis Software, v10.6.2 (Tree Star).
Cell population abundance	The C1QA+ cell proportions observed by FCM gradually decreased from 3 d (9.3±0.3%) until later stages (3 w: 8.8±0.5%, 16 w: 8.0±0.6%, and 78-82 w: 6.8±0.5%). The percentage of THY1+ cells in TMJ discs was 12.4±2.0% at 3 d and was dramatically decreased to <6% in the adult and aged stages.
Gating strategy	The FSC vs. SSC gating strategy is used to exclude cell debris and dead cells, which tends to have lower forward scatter levels and are found at the bottom left corner of the plot. Apoptotic cells tend to have lower FSC and higher SSC. FCS-A vs. FSC-H gating strategy is used to exclude doublets and to screen out single cells. Each test has a blank control which is used to screen out positive cells.

☒ Tick this box to confirm that a figure exemplifying the gating strategy is provided in the Supplementary Information.
